# Safety and Efficacy of Radiofrequency Ablation in the Management of Unresectable Bile Duct and Pancreatic Cancer: A Novel Palliation Technique

**DOI:** 10.1155/2013/910897

**Published:** 2013-04-08

**Authors:** Paola Figueroa-Barojas, Mihir R. Bakhru, Nagy A. Habib, Kristi Ellen, Jennifer Millman, Armeen Jamal-Kabani, Monica Gaidhane, Michel Kahaleh

**Affiliations:** Division of Gastroenterology and Hepatology, Department of Medicine, Weill Cornell Medical College, New York, NY 10021, USA

## Abstract

*Objectives*. Radiofrequency ablation (RFA) has replaced photodynamic therapy for premalignant and malignant lesions of the esophagus. However, there is limited experience in the bile duct. The objective of this pilot study was to assess the safety and efficacy of RFA in malignant biliary strictures. Methods: Twenty patients with unresectable malignant biliary strictures underwent RFA with stenting between June 2010 and July 2012. Diameters of the stricture before and after RFA, immediate and 30 day complications and stent patency were recorded prospectively. *Results*. A total of 25 strictures were treated. Mean stricture length treated was 15.2 mm (SD = 8.7 mm, Range = 3.5–33 mm). Mean stricture diameter before RFA was 1.7 mm (SD = 0.9 mm, Range = 0.5–3.4 mm) while the mean diameter after RFA was 5.2 mm (SD = 2 mm, Range = 2.6–9 mm). There was a significant increase of 3.5 mm (*t* = 10.8, DF = 24, *P* value = <.0001) in the bile duct diameter post RFA. Five patients presented with pain after the procedure, but only one developed mild post-ERCP pancreatitis and cholecystitis. Conclusions: Radiofrequency ablation can be a safe palliation option for unresectable malignant biliary strictures. A multicenter randomized controlled trial is required to confirm the long term benefits of RFA and stenting compared to stenting alone.

## 1. Introduction

Self-expanding metal stents (SEMS) have become the mainstay palliative treatment for malignant biliary obstruction in patients with a life expectancy greater than 3 months [1, 2]. Their use has improved bile duct patency beyond what was achieved with plastic stents; however, long-term patency continues to be an unresolved issue. SEMS can occlude from tissue ingrowth or overgrowth, benign epithelial hyperplasia or secondary to biofilm, and sludge formation within the lumen of the stent [3]. Up to 50% of patients will have stent occlusion in the first 6 to 8 months [4, 5]. Different design alternatives have been explored in an attempt to improve stent patency. Covered SEMS were designed to prevent tissue ingrowth; however, they are contra-indicated for hilar drainage, have higher migration rates, and might be associated with increased risks of pancreatitis and cholecystitis [6–11]. Another treatment strategy to prolong stent patency and eventual survival is photodynamic therapy (PDT). PDT showed promising results; however, it carries a high complication rate including cholangitis and photosensitivity requiring the patient to avoid direct exposure to light for 4–6 weeks [12–14]. 

Radiofrequency ablation (RFA) has been used for tumor ablation in the esophagus [15], rectum [16], and liver [17]. It utilizes heat to achieve contact coagulative necrosis of surrounding tissue. Within the bile duct it seems to lead to improved stent patency by decreasing tumor ingrowth and benign epithelial hyperplasia [18]. This technique has been widely used to treat primary and secondary liver cancer [17]; however, the experience in malignant biliary obstruction is limited. There have been animal studies to assess the power and duration of treatment [19], but there is only one study assessing this procedure in humans [20]. We aimed to assess the safety and efficacy of this novel palliative technique prospectively.

## 2. Methods

Data on twenty patients were collected between June 2010 and July 2012. Inclusion criteria included patients with unresectable malignant biliary strictures, unresectable cholangiocarcinoma, or pancreatic cancer with biliary obstruction and a life expectancy greater than 3 months. Exclusion criteria included cardiac pacemaker, instability for endoscopy, uncorrected coagulopathy, and pregnancy. Patients were evaluated with comprehensive laboratory studies as well as cross-sectional imaging prior to RFA and 30-days post RFA. All patients underwent RFA with either plastic or metal stent placement. Our primary outcome measures were the safety and efficacy of RFA. For efficacy measures, diameters of the stricture before and after RFA were recorded, as well as data on stent patency after a month was collected. Immediate and 30-day complications and stent patency were also recorded. Our study's primary endpoints were success rate—efficacy of RFA in terms of biliary stricture dilation and safety profile with respect to frequency and intensity of adverse events. The study was approved by the institutional ethics review committee (http://www.clinicaltrials.gov/ identifier NCT01303159). 

### 2.1. Technique of RFA

**Figure 1:**
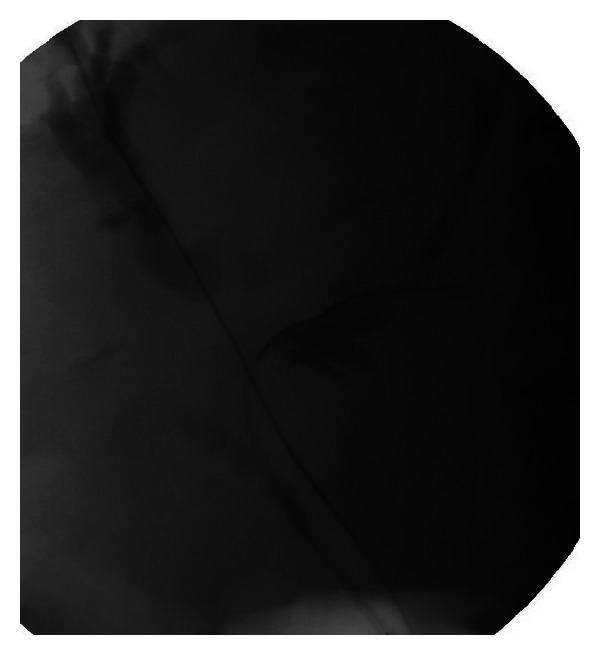
Fluoroscopic images of bile duct cancer at the confluence with a Bismuth III lesion.

**Figure 2:**
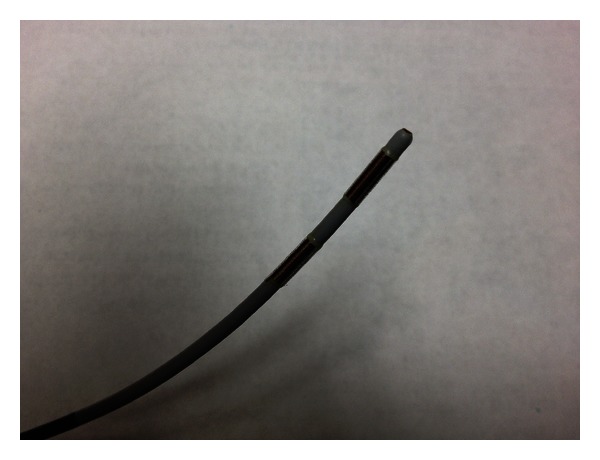
EndoHPB Probe for radio frequency ablation.

**Figure 3:**
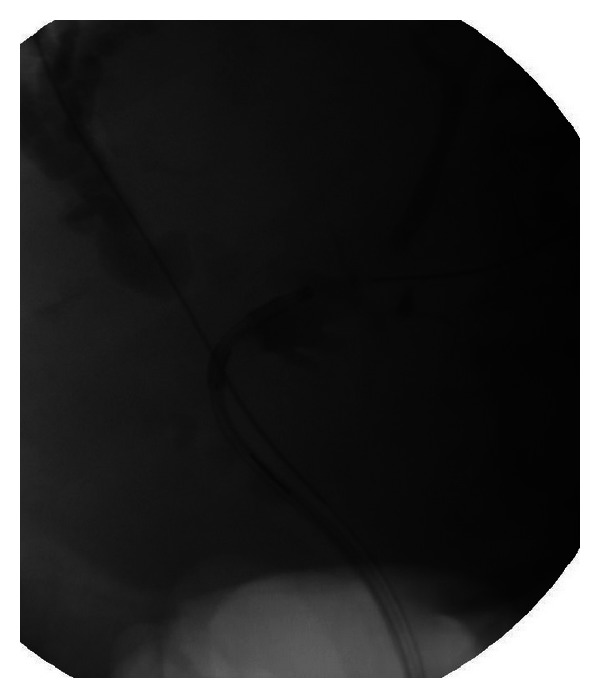
Application of radiofrequency at the level of the left hepatic duct.

**Figure 4:**
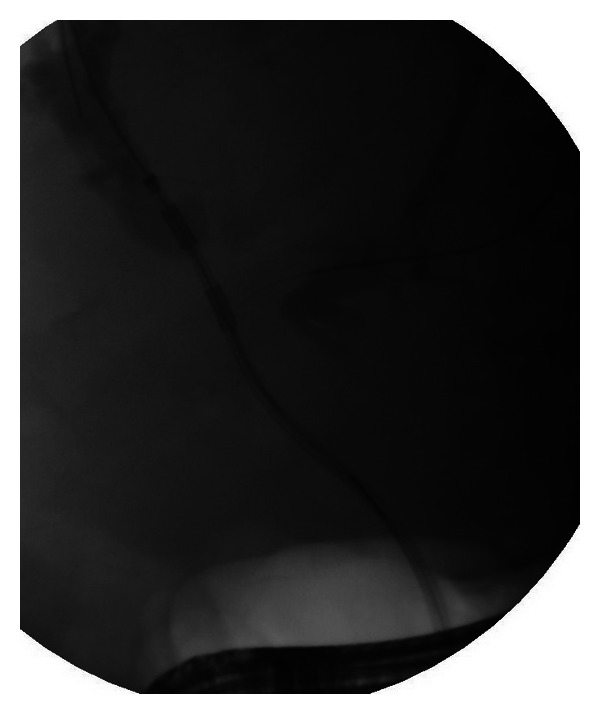
Application of radiofrequency ablation at the level of the right hepatic duct.

**Figure 5:**
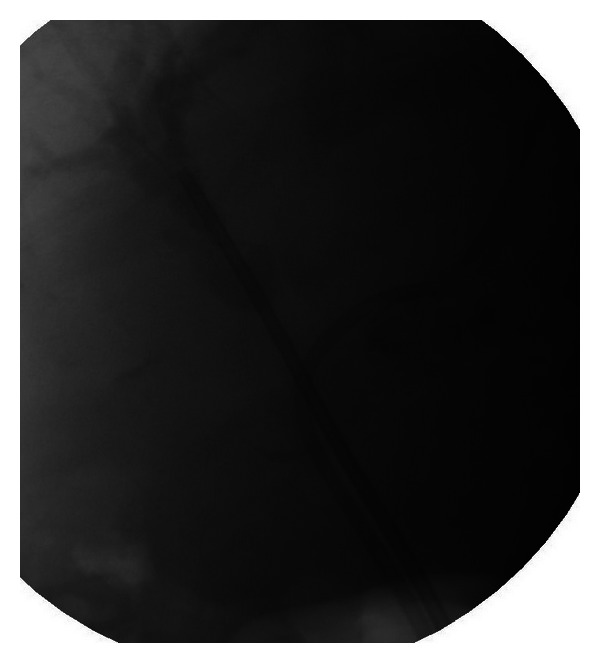
Placement of biliary stent posts radiofrequency application.

All procedures were performed under general anesthesia. Side viewing endoscopes TJF-160 and TJVF-160 (Olympus America, Center Valley, PA) were used for all procedures. All patients underwent biliary sphincterotomy. A cholangiogram was then performed to define stricture length and diameter (Figure 1). The Habib EndoHPB wire guided catheter (EMcision, Hitchin Herts, UK) was advanced over a wire at the level of the biliary stricture and ablation using a RITA 1500X RF generator (Angiodynamics, Latham, NY) set at 7–10 watts for a time period of 2 minutes was conducted (Figures 2, 3, and 4). A one-minute resting period after energy delivery was allowed before moving the catheter. Biliary stents were placed systematically after radiofrequency ablation (Figure 5). Immediate and 30-day complications as well as technical and intraprocedural difficulties were recorded. SAS 9.2 was used to conduct statistical analyses.

## 3. Results

Twenty patients (15 males) with a mean age of 65.3 years (range 45–86) were included in the study. A total of 25 malignant biliary strictures were treated with RFA. 11 patients had unresectable cholangiocarcinoma, 7 had unresectable pancreatic cancer, 1 had Intraductal papillary mucinous neoplasm (IPMN) with high grade dysplasia, and 1 had gastric cancer with metastatic tumor in the bile duct. Patient demographics are shown in Table 1. Deployment and application of the Habib EndoHPB catheter was successful in all 20 patients. The median stricture length treated was 9.7 mm. The mean stricture length treated was 15.2 mm (SD = 8.7 mm, Range = 3.5–33 mm). The mean stricture diameter before RFA was 1.7 mm (SD = 0.9 mm, Range = 0.5 − 3.4 mm) while the mean diameter after RFA 5.2 mm (SD = 2 mm, Range = 2.6–9 mm). The before and after RFA treated stricture diameters were compared using paired *t*-test. There was a significant increase of 3.5 mm (*t* = 10.8, DF = 24, *P* value ≤.0001) in the bile duct diameter after RFA. 

All patients were stented after the procedure. Patients undergoing repeated sessions of RFA received plastic stents. Patients receiving a single session of RFA were offered metal stents. Covered metal stents were placed for distal CBD strictures while uncovered metal stents were placed for hilar lesions. Covered self-expanding metal stents (SEMS) (Wallflex, Boston Scientific, Natick, MA) were used in 13 patients, 1 patient received an uncovered Wallflex SEMS and 6 patients received plastic stents. One of the patients with plastic stents received uncovered Wallflex stent at his second RFA session (Table 2). 

Three patients underwent choledochoscopy confirming tissue necrosis and ablation after RFA, one of them at three months after RFA just before his second session of RFA. Immediate and 30-day complications were collected for all patients. All stents were patent at day 30 for all patients. Five patients presented with pain after the procedure, but only one developed mild post-ERCP pancreatitis managed conservatively and cholecystitis which was drained percutaneously. 

## 4. Discussion

Endoscopic biliary decompression has become the preferred palliation technique in unresectable malignant biliary obstruction. Long-term biliary drainage continues to be a challenge and modifications to stent design have not proven to be an effective solution [3, 4, 11]. SEMS have been shown to offer longer palliation than plastic stents but can occlude due to tumor growth, sludge or biofilm formation. In patients with cholangiocarcinoma, PDT has shown improvement in overall survival; however, it is also associated with complications such as cholangitis, hemobilia, and photosensitivity (12.5–30%) [13, 14].

Initial experience with RFA comes from the Hammersmith team [20] and is very encouraging. In their study, they treated a total of 22 patients and demonstrated immediate and 30-day safety and 90-day biliary patency. They reported improved stricture after RFA treatment, with similar complication rates as in our study (1 asymptomatic biochemical pancreatitis, 2 cholecystitis) and one failure to decompress the bile duct, which eventually resulted in the patient's death [20]. 

Our study shows that RFA treatment of malignant biliary obstructions can be safe and effective. The complications described in animal models, including extension of the RFA burn into adjacent structures and difficult catheter reintroduction after treatment were not observed in this study [19]. The placement and application of the RFA probe was successful in 100% of our patients and there were no difficulties introducing other catheters after treatment for stent deployment. The coagulative necrosis induced by the probe has an immediate effect as confirmed by choledochoscopy and seems to be related to the intensity of the energy liberated by the probe in contact with the tumor; that is, the tighter the stricture, the more intense the contact and the amount of energy liberated. 

Five of the patients had postprocedural complications with only one being severe (i.e., cholecystitis requiring percutaneous drainage); however we remained within the expected post-ERCP/stent placement complications rate. These complications are primarily attributed to ERCP and/or stenting post RFA. All complications resolved with medical management and none required surgery.

Radiofrequency ablation seems to be an efficient and safe treatment strategy in palliation of unresectable malignant biliary obstructions. A prospective study, preferably randomized control trial, is required to confirm the benefits of RFA on long-term biliary stent patency and survival rates. 

## Figures and Tables

**Table 1:** Patient demographics.

Number of patients	20
Age y, median (range)	65.3 (45–86)
Sex (male/female)	15/5
Disease:	
Cholangiocarcinoma	11
Pancreatic cancer	7
IPMN	1
Gastric cancer	1

**Table 2:** Treatment results.

Number of strictures treated	25
Mean stricture length (mm)	15.2 (SD = 8.7, range = 3.5–33)
Mean stricture diameter before RFA (mm)	1.7 (SD = 0.9, range = 0.5–3.4)
Mean stricture diameter after RFA (mm)	5.2 (SD = 2, range = 2.6–9)
Type of stent placed	
Uncovered SEMS*	1
Partially or fully covered SEMS	13
Plastic stents	6
Complications	
Pain	5
Mild after ERCP pancreatitis	1
Cholecystitis	1

*SEMS: self-expanding metal stent.

## References

[1] Andersen JR, Sorensen SM, Kruse A, Rokkjaer M, Matzen P (1989). Randomised trial of endoscopic endoprosthesis versus operative bypass in malignant obstructive jaundice. *Gut*.

[2] Shepherd HA, Royle G, Ross APR, Diba A, Arthur M, Colin-Jones D (1988). Endoscopic biliary endoprosthesis in the palliation of malignant obstruction of the distal common bile duct: a randomized trial. *British Journal of Surgery*.

[3] Davids PHP, Groen AK, Rauws EAJ, Tytgat GNJ, Huibregtse K (1992). Randomised trial of self-expanding metal stents versus polyethylene stents for distal malignant biliary obstruction. *The Lancet*.

[4] O'Brien S, Hatfield ARW, Craig PI, Williams SP (1995). A three year follow up of self expanding metal stents in the endoscopic palliation of longterm survivors with malignant biliary obstruction. *Gut*.

[5] Rossi P, Bezzi M, Rossi M (1994). Metallic stents in malignant biliary obstruction: results of a multicenter European study of 240 patients. *Journal of Vascular and Interventional Radiology*.

[6] Kahaleh M, Tokar J, Conaway MR (2005). Efficacy and complications of covered Wallstents in malignant distal biliary obstruction. *Gastrointestinal Endoscopy*.

[7] Yoon WJ, Lee JK, Lee KH (2006). A comparison of covered and uncovered Wallstents for the management of distal malignant biliary obstruction. *Gastrointestinal Endoscopy*.

[8] Hatzidakis A, Krokidis M, Kalbakis K, Romanos J, Petrakis I, Gourtsoyiannis N (2007). ePTFE/FEP-covered metallic stents for palliation of malignant biliary disease: can tumor ingrowth be prevented?. *CardioVascular and Interventional Radiology*.

[9] Isayama H, Komatsu Y, Tsujino T (2004). A prospective randomized study of “covered” versus “uncovered” diamond stents for the management of distal malignant biliary obstruction. *Gut*.

[10] Suk KT, Kim HS, Kim JW (2006). Risk factors for cholecystitis after metal stent placement in malignant biliary obstruction. *Gastrointestinal Endoscopy*.

[11] Loew BJ, Howell DA, Sanders MK (2009). Comparative performance of uncoated, self-expanding metal biliary stents of different designs in 2 diameters: final results of an international multicenter, randomized, controlled trial. *Gastrointestinal Endoscopy*.

[12] Ortner MEJ, Caca K, Berr F (2003). Successful photodynamic therapy for nonresectable cholangiocarcinoma: a randomized prospective study. *Gastroenterology*.

[13] Pereira SP, Ayaru L, Rogowska A, Mosse A, Hatfield ARW, Bown SG (2007). Photodynamic therapy of malignant biliary strictures using meso-tetrahydroxyphenylchlorin. *European Journal of Gastroenterology and Hepatology*.

[14] Zoepf T, Jakobs R, Arnold JC, Apel D, Riemann JF (2005). Palliation of nonresectable bile duct cancer: improved survival after photodynamic therapy. *American Journal of Gastroenterology*.

[15] Khorsandi SE, Zacharoulis D, Vavra P (2008). The modern use of radiofrequency energy in surgery, endoscopy and interventional radiology. *European Surgery*.

[16] Vavra P, Dostalik J, Zacharoulis D, Khorsandi SE, Khan SA, Habib NA (2009). Endoscopic radiofrequency ablation in colorectal cancer: initial clinical results of a new bipolar radiofrequency ablation device. *Diseases of the Colon and Rectum*.

[17] Sutherland LM, Williams JAR, Padbury RTA, Gotley DC, Stokes B, Maddern GJ (2006). Radiofrequency ablation of liver tumors: a systematic review. *Archives of Surgery*.

[18] Steel AW, Postgate AJ, Vivianos P (2010). The use of a novel endoscopically placed radiofrequency probe for the management of malignant bile duct obstruction. *Gastrointestinal Endoscopy*.

[19] Khorsandi SE In vivo experiments for the development of a novel bipolar radiofrequency probe (EndoHPB) for the palliation of malignant biliary obstruction.

[20] Steel AW, Postgate AJ, Khorsandi S (2011). Endoscopically applied radiofrequency ablation appears to be safe in the treatment of malignant biliary obstruction. *Gastrointestinal Endoscopy*.

